# Association of Serum Uric Acid with cardio-metabolic risk factors and metabolic syndrome in seafarers working on tankers

**DOI:** 10.1186/s12889-020-08466-2

**Published:** 2020-04-05

**Authors:** Fereshteh Baygi, Kimmo Herttua, Ali Sheidaei, Alireza Ahmadvand, Olaf Chresten Jensen

**Affiliations:** 1 Centre of Maritime Health and Society, Department of Public Health, University of Southern Denmark, Esbjerg, Denmark; 2 Department of Biostatistics, Faculty of Paramedical Sciences, Shahid Beheshti University of Medical Sciences, Tehran, Iran; 3 School of Clinical Sciences, Faculty of Health, Queensland University of Technology, QLD, Brisbane, Australia

**Keywords:** Cardiometabolic, Risk factors, Metabolic syndrome, Sailors, Uric acid

## Abstract

**Background:**

There is still controversy over the clinical interpretation of the association between metabolic syndrome (MetS) and serum uric acid (SUA) levels. Therefore, the aim of this study was to investigate the association of SUA levels with MetS and other cardio-metabolic risk factors (CMRF) in seafarers working on tankers.

**Methods:**

This cross-sectional study was conducted in 2015 and included 234 male seafarers working on tankers. The participants were divided into three groups based on the tertiles of SUA. The report from of the National Committee of Obesity was used to define the MetS. The relationship between SUA, CMRF and MetS adjusted for age, educational level, job history, shift work, smoking and BMI was assessed by logistic regression analysis.

**Results:**

The subjects were aged 36.0 ± 10.3 years (mean ± SD). A notable upward trend was observed in mean weight, body mass index (BMI), triglyceride (TG), total cholesterol (TC), low density lipoprotein (LDL) and very low-density lipoprotein (VLDL) as tertiles of SUA increased (*P* < 0.001). In all models of the logistic regression analyses, the odds ratio (OR) of high TG for participants in the 3rd tertile of SUA was four times higher than that for participants in the 1st tertile of SUA (*P* < 0.001). The odds ratio of high TC and the SUA levels increased, so that the odds ratio of high TC for participants in the 2nd tertile was 2.47 (95% CI: 1.10–5.53) (*P* < 0.05) as compared with that for participants in the 1st tertile. Significant association was observed between MetS and the levels of SUA; 6.10 (95% CI: 1.77–20.94) (*P* < 0.05).

**Conclusions:**

Findings revealed that SUA levels were associated with MetS, high TG and high TC. Therefore, it is recommended that clinical attention should be given to symptoms related to elevated SUA - being one of the most important remediable risk factors for MetS - in the annual medical examinations of seafarers.

## Background

Metabolic syndrome (MetS) is a cluster of risk factors for major chronic diseases, including cardiovascular diseases (CVD), type II diabetes and hypertension. MetS can have serious adverse effects on overall health of individuals [1, 2]. It has been well established that MetS is associated with an increased risk of developing the CVD [3].

Different population groups have shown varied prevalence of the MetS based on several factors such as nationality, ethnic differences, or syndrome criteria. However, according to published studies, the MetS prevalence rates have increased all over the world in the past two decades [4, 5]. Recent studies have demonstrated that the MetS prevalence is 25.9 and 15.0% among Danish seafarers and Iranian male seafarers, respectively [1, 6]. The above figures in general population vary from 8.0% in India to 24.0% in USA and 7.0% in France to 43% in Iran, respectively [7]. Accordingly, the early detection of MetS risk factors is essential to take preventive actions.

Some clinical markers including serum uric acid (SUA), the count of white blood cells (WBC), and the level of alanine aminotransferase (ALT) have been identified as the probable risk factors for developing MetS [8, 9]. Even individuals with high normal levels of all three markers are at a higher risk of developing MetS [10–12].

Uric acid exerts a pro-inflammatory effect on endothelial cells which may be associated with MetS risk factors such as elevated triglyceride (TG) levels, hypertension and insulin resistance [13]. Moreover, in recent years, elevated SUA levels in adults have been suggested as CVD risk factors in some studies [14, 15]. It is not clear whether elevated SUA levels should be considered as independent risk factors or as a simple marker that reflect the association between uric acid and other MetS risk factors [16]. Also, the clinical interpretation of uric acid is still controversial because some studies conducted on adolescents suggest that the association between MetS and SUA may be gender-specific or limited to higher levels of SUA [17, 18]. The prevalence of Mets was high among seafarers [1, 6]. On the other hand, the presence of Mets is associated with the development of CVD and diabetes mellitus [19]. Elevated uric acid may turn out to be one of the more important remediable risk factors for MetS and cardiovascular diseases.

Health- related risk factors have an effect, not only on the health of the seafarer, but on economy, environment and public safety. So, identifying high-risk asymptomatic individuals with the MetS can lead to prevention and treatment of the subsequent cardiovascular events. This cross-sectional study aimed to evaluate the potential association between SUA levels and cardio-metabolic risk factors (CMRF) and MetS in seafarers working on tankers.

## Methods

### Study design and population

This cross-sectional study was conducted on 234 male seafarers working on ocean-going tankers of a shipping company. Sampling was performed from April to September 2015. A well-equipped health unit was located in the shipping company. According to the health policies of the shipping company, all seafarers who had at least 6 months of sea service had been referred to the health unit for annual medical examinations. Routinely, medical examinations were performed there by trained employees of the health unit according to standard protocols. Having at least 6-month sea service were considered as inclusion criteria, since seafarers’ medical data is not registered before that. All eligible male seafarers who were referred to the health unit of the company for routine annual medical examination during the study period were invited to participate in the study. They were explained that their medical data would be used for the current research and written informed consent was obtained from all participants. During the study period, 17 subjects with incomplete medical data were excluded from all analyses.

### Sample size

The main focus of this study was on the calculation of odds ratio from logistic regression analysis. Therefore, we used the formula proposed by Hsieh et al. (1998) [20]. The power and significance level were set to 0.8 and 0.05, respectively. In the previous study, the reported prevalence of Hypercholesterolemia was 23% among adult males [21]. In addition, we calculated odds ratios equally and more than 1.5 as the effect size. In this regard, the sample size was 212 adult male seafarers. We also added 10% to the sample size to cover the effect of missing and non-response error. Therefore, the final sample size was 234.

### Measurement

Weight, height, blood pressure (BP), waist circumferences (WC) and biomedical indicators such as fasting blood sugar (FBS), serum level of triglyceride, total cholesterol (TC), high density lipoprotein cholesterol (HDL-C) and SUA were measured. Detailed information regarding the procedures of measurement has been described previously [1].

MetS was diagnosed according to recommendation of the National Committee of Obesity [22]. Subjects who met at least three of following criteria were considered as patients with Mets: Abdominal obesity (WC > 95 cm), high TG level (> 150 mg/dl), low HDL-C level (< 40 mg/dl), elevated systolic blood pressure (SBP) > 130 mmHg and/ or elevated diastolic blood pressure (DBP) > 85 mmHg, and high FBS > 100 mg/dl.

Excess weight, high TC and high LDL-C level (low density lipoprotein cholesterol level) were considered as other CMRF. Excess weight was defined as body mass index (BMI) > 25 kg/m2. High TC and LDL were defined as follow: TC ≥ 200 mg/dl and LDL-C ≥ 130 mg/dl.

### Statistical analysis

Statistical analysis was done by using SPSS (Statistical Package for the Social Sciences software, version 21) at a significant level of 0.05. Continuous variables were expressed as mean ± standard deviation. Categorical variables were presented as numbers (percentage). Normality of continuous variables was examined by Kolmogorov-Smirnov test.

The subjects were divided into tertiles based on serum uric acid concentration. One-way analysis of variance (ANOVA) was used to compare the mean of CMRF across tertiles of SUA. Logistic regression was used to examine the association between CMRF and SUA. The following models were run in the logistic regression analysis: Model 1, crude model (without adjustment), Model 2: adjusted for age, educational level, job history, shift work, smoking status, and Model 3 additionally adjusted for BMI in all abnormalities except for excess weight.

## Results

The subjects were aged 36.0 ± 10.3 years (mean ± SD). Table 1 summarizes the characteristics of the study population according to tertiles of SUA.

**Table 1:** Characteristics of the participants by tertiles of serum uric acid

Parameter	Serum Uric Acid^a^			
	Tertile 1	Tertile 2	Tertile 3	***P***- value†
**Mean age (year)**	34.4 ± 9.8	37.2 ± 10.9	36.6 ± 10.0	0.21
**Educational level**				
Diploma	24 (29.3)^b^	25 (33.3)	22 (28.6)	0.78
Academic	58 (70.7)	50 (66.7)	55 (71.4)	
**Job history**				
= < 10 year	56 (68.3)	41 (54.7)	45 (58.4)	0.19
> 10 year	26 (31.7)	34 (45.3)	32 (41.6)	
**Shift work**				
No	22 (26.8)	26 (34.7)	22 (28.6)	0.53
Yes	60 (73.2)	49 (65.3)	55 (71.4)	
**BMI category**				
Normal	44 (53.7)	36 (48.0)	34 (47.2)	0.58
Overweight	34 (41.5)	31 (41.3)	34 (47.2)	
Obese	4 (4.9)	8 (10.7)	4 (5.6)	
**Smoking status**				
No	61 (74.4)	53 (70.7)	55 (71.4)	0.85
Yes	21 (25.6)	22 (29.3)	22 (28.6)	
**Total**	82 (35.0)	75 (32.0)	77 (32.9)	

*BMI* body mass index

^a^The ranges of serum uric acid levels are 1.6 mg/dl to 4.7 mg/dl for the 1st tertile, 4.7 mg/dl to 5.9 mg/dl for the 2nd tertile, and 5.9 mg/dl to 8.5 mg/dl for the 3rd tertile

**†***P*-value of ANOVA for age and chi square for other variables

^b^Percent are shown in parenthesis

Table 2 demonstrates the mean values of CMRF by tertiles of SUA. Mean weight, BMI, TG, TC, LDL and very low-density lipoprotein (VLDL) showed a significant increasing trend in the tertiles of SUA. Also, mean waist circumference and mean FBS were different in tertiles of SUA (with the *p*-value of 0.03 for both), but there was no linear trend.

**Table 2:** Mean values for cardiometabolic risk factors by tertiles of serum uric acid

Parameter Mean(±SD)	Serum Uric Acid^a^			
	Tertile 1	Tertile 2	Tertile 3	***P***- value
Weight (kg)	76.8 ± 11.4	80.9 ± 11.7	81.3 ± 11.5	0.02
Height (cm)	176.6 ± 6.3	178.1 ± 6.8	177.0 ± 6.7	0.38
WC (cm)	88.6 ± 10.6	92.5 ± 10.4	91.9 ± 10.2	0.03
BMI (kg/m^2^)	24.5 ± 3.1	25.5 ± 3.3	25.9 ± 3.3	0.02
SBP (mmHg)	120.5 ± 12.7	123.3 ± 12.9	124.3 ± 12.4	0.15
DBP (mmHg)	77.3 ± 9.3	78.9 ± 8.5	78.2 ± 7.6	0.50
FBS (mg/dl)	91.3 ± 23.4	98.9 ± 19.8	96.1 ± 9.3	0.03
TG (mg/dl)	104.0 ± 56.8	122.7 ± 67.7	153.8 ± 80.3	< 0.001
TC (mg/dl)	164.0 ± 40.5	186.1 ± 41.5	192.7 ± 37.6	< 0.001
HDL (mg/dl)	45.6 ± 11.4	47.1 ± 9.2	45.5 ± 9.4	0.52
LDL (mg/dl)	97.6 ± 31.1	114.0 ± 32.1	116.8 ± 32.1	< 0.001
VLDL (mg/dl)	20.6 ± 11.4	24.7 ± 13.5	30.6 ± 16.5	< 0.001

*WC* waist circumferences, *BMI* body mass index, *SBP* systolic blood pressure, *DBP* diastolic blood pressure, *FBS* fasting blood sugar, *TG* triglycerides, *TC* total cholesterol, *HDL-C* high-density lipoprotein cholesterol, *LDL-C* low-density lipoprotein cholesterol, *VLDL* Very low-density lipoprotein

^a^The ranges of serum uric acid levels are 1.6 mg/dl to 4.7 mg/dl for the 1st tertile, 4.7 mg/dl to 5.9 mg/dl for the 2nd tertile, and 5.9 mg/dl to 8.5 mg/dl for the 3rd tertile

We compared the distribution of cardio metabolic risk factors and Metabolic Syndrome among tertiles of serum uric acid. The prevalence of high TG, high LDL, high TC and MetS was 55.9, 43.5, 47 and 57.1% respectively in tertile 3, which was significantly higher than the other 2 groups. In addition, there is a positive significant relation between the number of MetS components and the serum uric acid components. In this regard, individuals with more MetS components are more likely to be in the third tertile of serum uric acid.

Table 3 shows the association between CMRF and the levels of SUA in the logistic regression analysis. In all models, the odds ratio of high TG for participants in the 3rd tertile of SUA was four times higher than that for participants in the 1st tertile of SUA (*P* < 0.001). The odds ratio of high TC and the level of SUA increased, so that the odds ratio of high TC for participants in the 2nd tertile was 2.47 (95% CI: 1.10–5.53) (*P* < 0.05) as compared with that for participants in the 1st tertile. Mentioned figure for subjects in the 3rd tertile was 3.77 (95% CI:1.72–8.27) (*P* < 0.05) compared to subjects of the first tertile. Also, a strong significant association was found between MetS and the levels of SUA; 6.10 (95% CI: 1.77–20.94) (*P* < 0.05).

**Table 3:** Association of cardiometabolic risk factors and serum uric acid in Logistic regression analysis^a^

Parameter	Serum Uric Acid^b^		
	Tertile 1	Tertile 2	Tertile 3
**Excess weight**			
Model 1	1 (reference)	1.25 (0.67–2.35)	1.46 (0.78–2.74)
Model 2	1 (reference)	1.13 (0.58–2.18)	1.42 (0.74–2.72)
**High TG**			
Model 1	1 (reference)	1.33 (0.57–3.1)	4.37 (2.04–9.36)
Model 2	1 (reference)	1.11 (0.45–2.72)	4.55 (2.03–10.22)
Model 3	1 (reference)	1.04 (0.42–2.57)	4.17 (1.84–9.45)
**High LDL**			
Model 1	1 (reference)	1.62 (0.76–3.46)	2.41 (1.16–5.00)
Model 2	1 (reference)	1.58 (0.73–3.43)	2.48 (1.18–5.21)
Model 3	1 (reference)	1.56 (0.71–3.39)	2.41(1.13–5.11)
**High TC**			
Model 1	1 (reference)	2.58 (1.18–5.66)	3.93 (1.83–4.43)
Model 2	1 (reference)	2.58 (1.16–5.74)	4.12 (1.89–8.94)
Model 3	1 (reference)	2.47 (1.10–5.53)	3.77 (1.72–8.27)
**Having MetS**			
Model 1	1 (reference)	3.35 (1.01–11.03)	6.84 (2.21–21.10)
Model 2	1 (reference)	2.89 (0.82–10.10)	7.45 (2.24–24.73)
Model 3	1 (reference)	2.44 (0.66–8.92)	6.10 (1.77–20.94)

*TG* triglycerides, *LDL-C* low-density lipoprotein cholesterol, *TC* total cholesterol, *Mets* metabolic syndrome

^a^Model 1 was without adjustment., Model 2 adjusted for age, educational level, job history, shift work, smoking status., and Model 3 additionally adjusted for body mass index in all abnormalities except for excess weight

^b^The ranges of serum uric acid levels are 1.6 mg/dl to 4.7 mg/dl for the1^st^ tertile, 4.7 mg/dl to 5.9 mg/dl for the 2nd tertile, and 5.9 mg/dl to 8.5 mg/dl for the 3rd tertile

## Discussion

In this study, we sought to assess the association of SUA with CMRF and MetS in seafarers who had undergone annual medical examination. Our results showed that the seafarers whose SUA was categorized in the 2nd and 3rd tertiles had significantly higher mean weight, WC, BMI, FBS, TG, TC, LDL and VLDL compared with their peers in the lower tertiles of SUA. A similar situation was observed in some studies conducted on adolescents which used tertiles or quartiles to categorize the population based on their SUA levels [17, 23]. According to a prospective study on adults, subjects with hyperuricemia had significantly higher BMI, TG and lower HDL-C. Also, a strong positive association among Insulin Resistant Syndrome (IRS) was observed between BMI and SUA levels [24].

A recent study conducted on elderly women showed that subjects in the second, third and fourth SUA quartiles had significantly higher risk of Mets in comparison with those in first uric acid quartile. But, after age- adjustment, there was no significant association between UA quartiles and all components of MetS among hypertensive subjects. This may be due to some existing residual confounding effects such as exercise, calorie and sodium intake, which were not included in the study [25].

A study on healthy adults revealed that the SUA level was higher in subjects with abnormal WC, TG, HDL and BP compared to those with normal levels. After adjusting for BMI which may be a confounding factor for SUA levels the influence of abnormal metabolic components on SUA levels decreased significantly. Also, elevated TG had the strongest effect on SUA levels [26].

The association between hypertension (HTN) and SUA has been explored a long time ago and the review studies showed an independent correlation between SUA and hypertension [27, 28].

In the present study, elevated SUA levels were associated with MetS, elevated TC and TG. However, there was no significant difference between SUA tertiles and the mean values of SBP and DBP. This controversy might be explained by following uncommon causes of hyperuricemia. However, we did not consider such confounders in our study. Renal disfunction [29, 30], small bowel diseases [31] and diet [32] have effects on clearance of uric acid without any especial effects on BP. People who suffer from kidney diseases have higher SUA [29]. On the other hand, 15% of uric acid clearance is through the gastrointestinal tract. So, small bowel diseases can increase SUA level without any effects on BP [30]. Some kinds of diets like the ones rich in fatty meat and seafood increase SUA levels as well [30]. A case-control study concluded that uric acid can be considered as a marker and potential modifier of MetS [33]. A study based on the health examination registration system data of the Taiwanese military service concluded that serum UA is an important predictor for the risk of incident of MetS, diabetes mellitus (DM), and HTN in adults, especially in males [34].

Consistent with our results, a study conducted in Iran demonstrated the association of SUA with Obesity, hypertriglyceridemia, hypertension and MetS [35]. Moreover, we found that after adjusting for confounding factors, there was a strong association between MetS and tertiles of SUA., so that in higher tertiles of SUA, the odds ratio of developing MetS was nearly six times higher. A Chinese cohort study showed that hyperuricemia was an independent risk factor for MetS in women, but it was not a significant risk factor for MetS and some of its components like TG and WC in men [36]. Other studies revealed that hyperuricemia is a risk factor for myocardial infractions and stroke [37], but the association of hyperuricemia with cardiometabolic risk factor has remained controversial so far. However, in some studies, uric acid is considered as an independent risk factor for MetS [38, 39]. A study conducted on Korean male workers revealed that risk of MetS was 1.6-fold in subjects with higher levels of uric acid in comparison with their peers in lower levels of SUA [40]. The Aerobics Center Longitudinal Study (ACLS) conducted on middle-aged and older subjects showed that there was a positive significant gradient between the incidence of MetS and SUA levels [41]. Another study revealed that individuals with high uric acid levels had higher odds of developing MetS [12]. Moreover, a recent study revealed that an increase in UA by 59 μmol/L over 7 years from the baseline led to an increase in odds of Mets of 28% [42].

Further support to our study comes from review studies which emphasize the hypothesis that hyperuricemia is a marker for MetS. One of the first review studies suggested that hyperuricemia may play a role in the development and pathogenesis of MetS, hypertension, stroke, and atherosclerosis [43].

In a meta-analysis, the researchers concluded that higher SUA levels led to an increased risk of MetS regardless of the study characteristics which were consistent with a linear dose-response relationship. In addition, SUA was a causal factor for the non-alcoholic fatty liver disease risk [44]. Srikanthan et al. in another review, found that different biomarkers like SUA were significantly correlated with MetS [45].

### Strength and limitations

First, because of the nature of cross-sectional studies, a causal relationship between SUA and cardiometabolic risk factors cannot be evaluated. Future studies are needed to address the longitudinal association between SUA concentration and Mets incidence. Second, we were not able to investigate dietary habits of subjects which may affect the serum uric acid concentration. Third, some information such as the history of gout or kidney disease was unavailable, which might have affected our results. To our knowledge, this is the first study investigating such relationships in seafarers.

## Conclusions

The results of the present study demonstrated that elevated SUA levels were associated with higher risk of MetS, high TG and high TC, which were in line with several studies. In annual medical examination of seafarers, physicians should pay attention to elevated SUA levels as a symptom of MetS and related risk factors, not as a sign of gout.

## Data Availability

The datasets generated and analyzed during the current study are not publicity available due to confidential policy of the shipping company but might be available with possible permission of the shipping company from corresponding author on reasonable request.

## Abbreviations

MetS: Metabolic Syndrome
SUA: Serum Uric Acid
CMRF: Cardio-Metabolic Risk Factors
BMI: Body Mass Index
TG: Triglyceride
TC: Total Cholesterol
LDL: Low Density Lipoprotein
VLDL: Very Low-Density Lipoprotein
mm Hg: Millimeters of Mercury
cm: Centimeter
mg: Milligram
m^2^: Square Meter
WBC: White Blood Cells
ALT: Alanine Aminotransferase
HDL-C: High Density Lipoprotein Cholesterol
SBP: Systolic Blood Pressure
DBP: Diastolic Blood Pressure
IRS: Insulin Resistant Syndrome
DM: Diabetes Mellitus
ACLS: Aerobics Center Longitudinal Study
